# Associations between dietary potassium intake and urinary potassium excretion: a protocol for systematic review and meta-analysis

**DOI:** 10.1186/s13643-024-02603-3

**Published:** 2024-07-17

**Authors:** Nobuhisa Morimoto, Hasan Jamil, Mohab Alakkari, Yuki Joyama, Tatsuhiko Anzai, Kunihiko Takahashi, Soichiro Iimori

**Affiliations:** 1 Department of Nephrology, Graduate School of Medical and Dental Sciences, Tokyo Medical and Dental University, Tokyo, 113-8519 Japan; 2 St. Luke’s International University, Tokyo, Japan; 3 Al-Baath University Hospital, Homs, Syria; 4 Columbia University Mailman School of Public Health, New York, USA; 5 Department of Biostatistics, Tokyo Medical and Dental University, M&D Data Science Center, Tokyo, Japan

**Keywords:** Nutrition and dietetics, Epidemiology, Chronic renal failure

## Abstract

**Background:**

While numerous studies have reported associations between low dietary potassium intake and adverse clinical outcomes, methods to estimate potassium intake, mainly self-reported dietary measures and urinary potassium excretion, entail certain limitations. Self-reported measures are subject to underreporting and overreporting. Urinary potassium excretion is affected by multiple factors including renal function. Revealing the degree of bias inherent in these measures would help accurately assess potassium intake and its association with disease risk. We aim to summarize evidence on the strength of the associations between potassium intake estimated from 24-h urinary potassium excretion and potassium intake estimated from self-reported dietary measures or objective quantification methods in populations with different kidney function levels and age groups. We also aim to identify factors that affect the association strength.

**Methods:**

We will search for potentially eligible studies that examined associations between self-reported potassium intake, 24-h urinary potassium excretion, and objectively quantified potassium intake, using MEDLINE (PubMed), Embase, Web of Science, and Scopus. Studies on children, adolescents, adults, and the elderly are eligible. Studies of patients on dialysis will be excluded. Collective study results, including a meta-analysis, will be synthesized if an adequate number of studies examining similar dietary potassium intake estimation methods are found. Analyses will be performed separately according to age groups and renal function. For the meta-analysis, fixed-effects or random-effect models will be employed depending on the degree of study heterogeneity to combine across studies the correlation coefficient, ratio, or standardized mean difference for potassium intake, comparing dietary potassium intake based on self-reported or objectively quantified methods and intake based on 24-h urinary potassium excretion. The degree of heterogeneity among included studies will be examined by calculating *I*^2^ statistics. To investigate sources of study heterogeneity, random-effects meta-regression analyses will be performed.

**Discussion:**

Revealing the strength of the association between dietary and urinary measures in populations with different levels of kidney function and age groups will enhance researchers’ and clinicians’ ability to interpret studies that utilize these measures and help establish a more solid evidence base for the role of potassium intake in changing chronic disease risk. Identifying factors that modify the associations between these measures may aid in developing predictive models to estimate actual potassium intake.

**Systematic review registration:**

PROSPERO CRD42022357847.

**Supplementary Information:**

The online version contains supplementary material available at 10.1186/s13643-024-02603-3.

## Background

Low potassium intake has been associated with an increased risk of chronic diseases, including hypertension, cerebrovascular disease, and chronic kidney disease (CKD) [1, 2]. Most prior studies relied on self-reported dietary measures or urinary potassium excretion to estimate dietary potassium intake [1, 2]. While self-reported dietary measures, such as a 24-h recall (24HR) and food frequency questionnaires (FFQ), are inexpensive with less participant burden, they are subject to bias due to overreporting and underreporting [3]. Twenty-four-hour urinary potassium excretion has been considered the gold standard for potassium intake estimation and has been used as a reference in validation studies of these self-reported measures [3, 4]. Pooled analyses of five large validation studies conducted in the US demonstrated a fairly good correlation between potassium intake estimated from self-report instruments, including 24HR and FFQ, and intake estimated from 24-h urinary potassium excretion [3]. However, validation studies performed in other countries and different age groups reported variable levels of associations between these measures [5–8]. Little is known about how the strength of associations between self-reported potassium intake and 24-h urinary potassium excretion changes in different populations.

While 24-h urinary potassium excretion likely captures short-term potassium intake more accurately than self-reported methods, various factors, including renal impairment and renin–angiotensin–aldosterone-system inhibitors, can alter urinary potassium excretion [2, 9, 10], thereby introducing bias to the urine-derived potassium intake. If researchers and clinicians are unaware of the possible bias inherent in urinary measures, they may misinterpret the association between dietary potassium intake and clinical outcomes as reported in previous studies. For example, several epidemiological studies showed the association between increased potassium intake based on increased urinary potassium excretion and reduced cardiovascular disease risk in patients with mild CKD [2]. While the findings suggest the possibility that increased potassium intake may be beneficial in lowering the cardiovascular disease risk of patients with CKD, increased urinary potassium excretion may simply reflect better renal function, which would be associated with lower cardiovascular disease risk [11]. Thus, an accurate understanding of the association strength between dietary potassium intake and urinary potassium excretion at different stages of CKD is important in applying the research findings to clinical practice. Few studies have systematically evaluated the strength of associations between urinary potassium excretion and objectively quantified dietary potassium intake at different stages of CKD and in different age groups. Revealing the strength of the association between dietary and urinary measures in different populations will enhance researchers’ and clinicians’ ability to interpret studies that utilize these measures. Identifying factors that modify the associations between these measures may also aid in developing predictive models to estimate actual potassium intake.

This protocol describes an ongoing systematic review and meta-analysis of studies that examined associations between self-reported potassium intake, 24-h urinary potassium excretion, and objectively quantified potassium intake.

### Research objectives and review questions

We aim to examine the strength of associations between potassium intakes estimated from self-reported or objectively quantified dietary measures and urinary potassium excretion data. We also aim to identify factors that affect the strength of the associations between these measures. Our specific review questions include the following:

- How strongly are self-reported dietary potassium intake and urinary potassium excretion correlated?
- How strongly are objectively measured dietary potassium intake and urinary potassium excretion correlated?
- What factors influence the strength of the correlation between dietary potassium intake and urinary potassium excretion?
- How do the correlations differ at different kidney function levels?

## Methods

This systematic review protocol is reported in line with the Preferred Reporting Items for Systematic Reviews and Meta-analysis Protocols (PRISMA-P) statement (Supplementary Table 1). The protocol is registered on the International Prospective Register of Systematic Reviews (PROSPERO) (CRD42022357847). This project is expected to be completed and published by 2024. Any changes to the protocol will be made on the PROSPERO.

### Eligibility criteria

#### Study designs

Cross-sectional studies that reported the association between potassium intakes based on self-reported or objectively measured dietary measures and urinary potassium excretion data are included. Longitudinal studies are also eligible if they reported these measures at one point in time.

#### Study population

Studies on children, adolescents, adults, and the elderly are eligible. Studies on infants (12 months or younger) will be excluded given the limited consumption of solid foods. Studies on individuals with CKD (defined as estimated glomerular filtration rate (eGFR) < 60 ml/min/1.73 m^2^ and/or persistent presence of markers of kidney damage, including albuminuria, hematuria, structural or functional kidney abnormalities, and a history of kidney transplantation [12]) not undergoing hemodialysis or peritoneal dialysis and studies on individuals without CKD are eligible. Studies on patients undergoing hemodialysis or peritoneal dialysis will be excluded as the amount of their urine is expected to be small. Studies on individuals with markedly low food intake (< 500 kcal/day) will be also excluded because of limited applicability to many individuals consuming a regular diet.

#### Intervention/exposure and comparators

This study will not evaluate interventions. However, we aim to compare potassium intake or potassium intake density (mg K/caloric intake) estimated from self-reported dietary measures or objective measurement and intake estimated from 24-h urinary potassium excretion. Therefore, the main exposure of interest is dietary potassium intake based on self-reported dietary measures or objective measurements. The comparator will be potassium intake estimated from 24-h urinary potassium excretion (recovery biomarker).

#### Outcomes and prioritization

The primary outcomes of this systematic review are dietary reporting error measures that include the following: (1) correlation coefficient between self-reported intake or objectively measured intake and 24-h urinary potassium excretion, (2) ratio of the mean (or median) self-reported or objectively quantified potassium intake to mean (or median) potassium intake estimated from 24-h urinary potassium excretion, and (3) mean difference between self-reported or objectively quantified potassium intake and potassium intake estimated from 24-h urinary potassium excretion or difference between log-reported intake and log-biomarker value.

The secondary outcomes of this systematic review are factors that affect the association strength between potassium intakes estimated from self-reported dietary measures or objectively quantified dietary measures and those estimated from 24-h urinary potassium excretion data.

#### Setting characteristics

There are no restrictions on the type of setting or location of the studies performed.

#### Language

Studies reported in English and Japanese are eligible based on the language proficiency of reviewers.

#### Information sources

Electronic databases, including MEDLINE (PubMed), Embase, Web of Science, and Scopus, are searched. The publication date is between January 1, 1950, and December 31, 2023. The search was performed on 5/12/2024. Reference lists of review articles will also be searched.

#### Search strategy for electronic databases

We performed scoping searches to refine our search strategy to retrieve appropriate references from the databases. Specifically, we validated our search strategy by checking if our search strategy could identify a pre-defined set of known articles using MEDLINE (PubMed). Our search strategy uses appropriate Boolean operators to combine the following keywords and Medical Subject Heading terms: potassium, urine, diet surveys, nutrition surveys, diet records, 24-h dietary recall, food frequency questionnaire, food weighing, feeding study, controlled diet, and dietary intake (Supplementary Table 2). Our final search strategy identified 100% of the pre-defined set of known articles using MEDLINE (PubMed) (Supplementary Table 3).

### Study records

#### Data management

Individual records are retrieved from MEDLINE (PubMed), Embase, Web of Science, and Scopus and are imported into an online systematic review tool called Rayyan (https://rayyan.ai/). Reviewers have access to a shared Rayyan account so that they can read the abstracts of retrieved studies.

#### Selection process

At least two independent reviewers will screen the titles and abstracts of studies found by our search strategy using the Rayyan software. If eligibility cannot be determined by titles or abstracts, the reviewers will screen full-text articles to see if they meet eligibility criteria. Disagreements will be resolved by discussion. If a consensus is not reached, a third reviewer will adjudicate.

#### Data collection process

We created a data extraction form using Microsoft Excel to collect relevant information from eligible studies (Supplementary Table 4).

### Data items

The following information will be extracted from eligible studies and stored in the data extraction form in Microsoft Excel: (1) study characteristics; (2) demographic information of study participants and sample size; (3) method of dietary potassium intake estimation (24HR, FFQ, weighing method, etc.); (4) method of urinary potassium excretion measurement (24-h urine collection) and method used to evaluate the completeness of 24-h urine collection; (5) dietary reporting error measures and their 95% confidence intervals ((a) coefficient of correlation between self-reported intake or objectively measured intake and 24-h urinary potassium excretion, (b) ratio of mean (or median) self-reported or objectively quantified potassium intake to mean (or median) potassium intake estimated from 24-h urinary potassium excretion, (c) mean difference between self-reported or objectively quantified potassium intake and potassium intake estimated from 24-h urinary potassium excretion or difference between log-reported intake and log-biomarker value); (6) potential confounders/covariates included in multivariable models; (7) definitions of CKD and parameters that reflect renal function; (8) the presence of comorbidities; and (9) medication use that may affect urinary potassium excretion.

### Risk of bias in individual studies

To address the risk of bias in included studies, we will employ Joanna Briggs Institute (JBI) tool for cross-sectional studies. The checklist consists of eight questions regarding inclusion criteria, exposure measurement, and outcome measurement (Supplementary Table 5). A single reviewer will assess the quality of individual studies based on these criteria.

### Statistical analyses

We will synthesize collective study results, including a meta-analysis if we find an adequate number of studies examining similar dietary potassium intake estimation methods. We will perform analyses by stratifying studies based on age groups and renal function, which may affect the strength of the associations between dietary potassium intake and urinary potassium excretion [13–15]. We will perform a meta-analysis if at least two studies with similar characteristics (i.e., patients with CKD) are found. If only one study is found, we will describe the results narratively. For the meta-analysis, we will use fixed-effects or random-effect models depending on the degree of study heterogeneity to combine across studies the correlation coefficient, ratio, or standardized mean difference for potassium intake, comparing dietary potassium intake based on self-reported or objectively quantified methods and intake based on 24-h urinary potassium excretion. The degree of heterogeneity among included studies will be examined by calculating *I*^2^ statistics. *I*^2^ statistics ≥ 30% will be considered to indicate some degree of heterogeneity [16]. To investigate sources of study heterogeneity, random-effects meta-regression analyses will be performed if at least 10 studies are included in the meta-analysis [16]. Specifically, the outcome variable will be the effect estimate, including a correlation coefficient, a ratio, and a standardized mean difference, and the explanatory variables will be participants’ age and renal function. The meta-regression analyses will be performed using the “metareg” command in the STATA statistical package.

### Subgroup analyses

We will perform subgroup analyses by stratifying studies based on the age and renal function of study participants.

#### Age group

Because previous studies indicated that age may affect the accuracy of dietary reporting [14, 15], we will seek to examine whether the association strength between self-reported dietary potassium intake and urinary potassium excretion differs by age group. The age cutoffs for age groups were determined based on the cutoffs used in previous systematic reviews as follows [17–19]:

(A) Children (1–9 years)
(B) Adolescents (10–18 years)
(C) Adults (19–64 years)
(D) Elderly (65 years or older)

We will perform a meta-analysis if at least two studies in the same age category (i.e., adults) are found. If only one study is found, we will describe the results narratively. In case the age range of an individual study overlaps in multiple age categories (e.g., 5–18 years), the study will be grouped into a separate group (e.g., children and adolescents). To avoid redundancy, such a study will not be included in the above four age groups. If at least two studies with similar overlapping age ranges are found, these studies will be combined, and a meta-analysis will be performed. Otherwise, a narrative review will be performed.

#### Renal function

We will also examine the association strength between dietary potassium intake and urinary potassium excretion in healthy participants and those with CKD, which could affect urinary potassium excretion [10] and likely weaken the strength of the association between dietary potassium intake and urinary potassium excretion. Because CKD includes a variety of diseases that may increase or decrease urinary potassium excretion and patients with CKD often take medications that may increase or decrease urinary potassium excretion [10, 13, 20], the assessment of the association between dietary potassium intake and urinary potassium excretion will likely be very complex. Considering the complexity of multiple factors that affect urinary potassium excretion in patients with CKD, we will stratify studies as follows:

(A) Individuals without any kidney diseases who are not taking any medications that may affect urinary potassium excretion
(B) Individuals without any kidney diseases who are taking medications that may affect urinary potassium excretion
(C) Individuals with CKD who have reduced eGFR
(D) Individuals with CKD who have normal eGFR but markers of kidney damage, including persistent albuminuria, hematuria, structural or functional kidney abnormalities, and a history of kidney transplantation

In this systematic review, we will stratify patients with CKD based on eGFR only and narratively review studies on specific renal diseases or other diseases that may affect urinary potassium excretion, which include the following:

- Patients with kidney diseases that may cause increased potassium excretion (i.e., renal tubular acidosis (types I and type II) and intrinsic renal transport defects) [13]
- Patients with kidney diseases that may cause decreased potassium excretion (i.e., primary renal tubular defects including systemic lupus erythematosus, obstructive uropathy, and hereditary tubular defects) [13]
- Patients with other comorbidities that may affect the associations between dietary potassium intake and urinary potassium excretion (i.e., hypoaldosteronism and primary hyperaldosteronism) [13]

In addition to kidney diseases, medication use is an important factor that could attenuate the strength of the association between dietary potassium intake and urinary potassium excretion because some medications may increase urinary potassium excretion (i.e., loop diuretics, thiazides, certain antimicrobials, mineralocorticoids, and glucocorticoids) [13, 20], while others may decrease urinary potassium excretion (i.e., angiotensin-converting enzyme inhibitors, angiotensin receptor blockers, potassium-sparing diuretics, nonsteroidal anti-inflammatory drugs, and calcineurin inhibitors) [20]. However, stratifying studies based on specific medications will be difficult especially for patients with CKD because some patients may take two or more medications that could affect urinary potassium excretion simultaneously (i.e., loop diuretics and angiotensin-converting enzyme inhibitors). Additionally, some studies may include both patients on medications that affect urinary potassium excretion and patients not on such medications. Therefore, we will stratify studies on individuals without CKD based on medication types, while for patients with CKD, we will narratively review medication types that could affect the association strength between dietary potassium intake and urinary potassium excretion.

### Meta-bias

Funnel plots will be created and visually inspected for asymmetry, which raises the possibility of the presence of publication bias [21]. When 10 or more studies are included in the meta-analysis, the Egger test will be conducted to evaluate the asymmetry of the funnel plots [21, 22].

### Patient and public involvement

No patient was involved.

### Confidence in cumulative evidence

As this systematic review will not evaluate interventions, Grading of Recommendations, Assessment, Development and Evaluations (GRADE) will not be implemented.

## Discussion

Our systematic review will summarize the evidence on the association strength between potassium intake based on self-reported instruments and urinary potassium excretion. Additionally, our review will aim to summarize the results of studies on the association between potassium intake estimated from urinary potassium excretion and objectively quantified potassium intake. The findings of our review will likely help researchers and clinicians interpret the results of epidemiological studies that evaluate the relationship between dietary potassium intake and disease risk more accurately. Our systematic review will also identify areas that require further research. For example, the association strength between dietary potassium intake and urinary potassium excretion in populations with altered urinary potassium excretion may not have been adequately investigated [2]. Ultimately, we aim to synthesize results that will guide future research and help improve the accuracy and precision of dietary assessment methods that estimate dietary potassium intake.

### Supplementary Information

Supplementary Material 1: Supplemental Table S1. PRISMA-P (Preferred Reporting Items for Systematic review and Meta-Analysis Protocols)  2015 checklist: recommended items to address in a systematic review protocol. Supplemental Table S2. Detailed search strategy. Supplemental Table S3. A list of a pre-defined set of articles used to validate our search strategy. Supplemental Table S4. Data extraction form. Supplementary Table S5. Joanna Briggs Institute critical appraisal checklist for analytical cross-sectional studies.

## Data Availability

The datasets used and/or analyzed during the current study are available from the corresponding author upon reasonable request.

## Abbreviations

CKD: Chronic kidney disease
24HR: 24-h recall
FFQ: Food frequency questionnaires
PROSPERO: International Prospective Register of Systematic Reviews
eGFR: Estimated glomerular filtration rate
PRISMA-P: Preferred Reporting Items for Systematic reviews and Meta-Analysis Protocols
JBI: Joanna Briggs Institute
GRADE: Grading of Recommendations, Assessment, Development and Evaluations
