# Supplementary material for: Associations between dietary potassium intake and urinary potassium excretion: a protocol for systematic review and meta-analysis
Source: Syst Rev. 2024 Jul 17;13:185. doi: 10.1186/s13643-024-02603-3 (PMC11253472; doi:10.1186/s13643-024-02603-3)
Supplement: Supplementary file 1 — Supplementary Material 1: Supplemental Table S1. PRISMA-P (Preferred Reporting Items for Systematic review and Meta-Analysis Protocols) 2015 checklist: recommended items to address in a systematic review protocol. Supplemental Table S2. Detailed search strategy. Supplemental Table S3. A list of a pre-defined set of articles used to validate our search strategy. Supplemental Table S4. Data extraction form. Supplementary Table S5. Joanna Briggs Institute critical appraisal checklist for analytical cross-sectional studies. [file 13643_2024_2603_MOESM1_ESM.docx]

**Supplemental Table S1.**

PRISMA-P (Preferred Reporting Items for Systematic review and Meta-Analysis Protocols) 2015 checklist: recommended items to address in a systematic review protocol*

| Section and topic | Item No | Checklist item | Reported on Page # |
| --- | --- | --- | --- |
| ADMINISTRATIVE INFORMATION | | |  |
| Title: |  |  |  |
| Identification | 1a | Identify the report as a protocol of a systematic review | 1 |
| Update | 1b | If the protocol is for an update of a previous systematic review, identify as such | Not applicable |
| Registration | 2 | If registered, provide the name of the registry (such as PROSPERO) and registration number | 2 |
| Authors: |  |  |  |
| Contact | 3a | Provide name, institutional affiliation, e-mail address of all protocol authors; provide physical mailing address of corresponding author | 1,6 |
| Contributions | 3b | Describe contributions of protocol authors and identify the guarantor of the review | 6 |
| Amendments | 4 | If the protocol represents an amendment of a previously completed or published protocol, identify as such and list changes; otherwise, state plan for documenting important protocol amendments | 3 |
| Support: |  |  |  |
| Sources | 5a | Indicate sources of financial or other support for the review | 6 |
| Sponsor | 5b | Provide name for the review funder and/or sponsor | Not applicable |
| Role of sponsor or funder | 5c | Describe roles of funder(s), sponsor(s), and/or institution(s), if any, in developing the protocol | Not applicable |
| INTRODUCTION | | |  |
| Rationale | 6 | Describe the rationale for the review in the context of what is already known | 2 |
| Objectives | 7 | Provide an explicit statement of the question(s) the review will address with reference to participants, interventions, comparators, and outcomes (PICO) | 2 |
| METHODS | | |  |
| Eligibility criteria | 8 | Specify the study characteristics (such as PICO, study design, setting, time frame) and report characteristics (such as years considered, language, publication status) to be used as criteria for eligibility for the review | 3 |
| Information sources | 9 | Describe all intended information sources (such as electronic databases, contact with study authors, trial registers or other grey literature sources) with planned dates of coverage | 3 |
| Search strategy | 10 | Present draft of search strategy to be used for at least one electronic database, including planned limits, such that it could be repeated | 3,  Supplementary Table S2 |
| Study records: |  |  |  |
| Data management | 11a | Describe the mechanism(s) that will be used to manage records and data throughout the review | 3 |
| Selection process | 11b | State the process that will be used for selecting studies (such as two independent reviewers) through each phase of the review (that is, screening, eligibility and inclusion in meta-analysis) | 3–4 |
| Data collection process | 11c | Describe planned method of extracting data from reports (such as piloting forms, done independently, in duplicate), any processes for obtaining and confirming data from investigators | 4,  Supplementary Table S4 |
| Data items | 12 | List and define all variables for which data will be sought (such as PICO items, funding sources), any pre-planned data assumptions and simplifications | 4 |
| Outcomes and prioritization | 13 | List and define all outcomes for which data will be sought, including prioritization of main and additional outcomes, with rationale | 3 |
| Risk of bias in individual studies | 14 | Describe anticipated methods for assessing risk of bias of individual studies, including whether this will be done at the outcome or study level, or both; state how this information will be used in data synthesis | 4  Supplementary Table S5 |
| Data synthesis | 15a | Describe criteria under which study data will be quantitatively synthesised | 4 |
|  | 15b | If data are appropriate for quantitative synthesis, describe planned summary measures, methods of handling data and methods of combining data from studies, including any planned exploration of consistency (such as I^2^, Kendall’s τ) | 4 |
|  | 15c | Describe any proposed additional analyses (such as sensitivity or subgroup analyses, meta-regression) | 4–5 |
|  | 15d | If quantitative synthesis is not appropriate, describe the type of summary planned | 4–5 |
| Meta-bias(es) | 16 | Specify any planned assessment of meta-bias(es) (such as publication bias across studies, selective reporting within studies) | 5 |
| Confidence in cumulative evidence | 17 | Describe how the strength of the body of evidence will be assessed (such as GRADE) | 5 |

*** It is strongly recommended that this checklist be read in conjunction with the PRISMA-P Explanation and Elaboration (cite when available) for important clarification on the items. Amendments to a review protocol should be tracked and dated. The copyright for PRISMA-P (including checklist) is held by the PRISMA-P Group and is distributed under a Creative Commons Attribution Licence 4.0.**

*From: Shamseer L, Moher D, Clarke M, Ghersi D, Liberati A, Petticrew M, Shekelle P, Stewart L, PRISMA-P Group. Preferred reporting items for systematic review and meta-analysis protocols (PRISMA-P) 2015: elaboration and explanation. BMJ. 2015 Jan 2;349(jan02 1):g7647.*

**Supplemental Table S2.**

Detailed search strategy

**MEDLINE (PubMed)** **via** [**https://pubmed.ncbi.nlm.nih.gov**](https://pubmed.ncbi.nlm.nih.gov/) (Search date: 5/12/2024; 737 results)

| #1 | "potassium"[Mesh] OR "potassium"[Tiab] |  |
| --- | --- | --- |
| #2 | "biomarkers/urine"[Mesh] OR "urine*"[Tiab] OR "urinary"[Tiab] OR "biomarker*"[Tiab] |  |
| #3 | "diet surveys"[Mesh] OR "diet survey*"[Tiab] OR "dietary survey*"[Tiab] OR "diet records"[Mesh] OR "diet record*"[Tiab] OR "dietary record*"[Tiab] OR "surveys and questionnaires"[Mesh] OR "nutrition surveys"[Mesh] OR "nutrition survey*"[Tiab] OR "nutritional survey*"[Tiab] OR "self-report"[Mesh] OR "self-report*"[Tiab] OR "dietary intake*"[Tiab] OR "diet intake*"[Tiab] OR "24-h dietary recall*"[Tiab] OR "24-hour dietary recall*"[Tiab] OR "24-hour diet recall*"[Tiab] OR "24-hour recall*"[Tiab] OR "food frequency questionnaire*"[Tiab] OR "FFQ"[Tiab] OR "FFQs"[Tiab] OR "food weighing"[Tiab] OR "weighed food*"[Tiab] OR "feeding stud*"[Tiab] OR "controlled diet*"[Tiab] OR "control diet*"[Tiab]) |  |
| #4 | 1950/1/1:2023/12/31[dp] |  |
| #5 | humans[Filter] |  |
| #6 | #1 AND #2 AND #3 AND #4 AND #5 |  |

**EMBASE via** [**https://www.embase.com**](https://www.embase.com) (Search date: 5/12/2024; 1782 results)

| #1 | 'potassium'/exp OR ('potassium'):ab,ti,kw |  |
| --- | --- | --- |
| #2 | 'urine'/exp OR ('urine*' OR 'urinary' OR 'biomarker*'):ab,ti,kw |  |
| #3 | 'dietary intake'/exp OR ('diet survey*' OR 'dietary survey*' OR 'diet record*' OR 'dietary record*' OR 'nutrition survey*' OR 'nutritional survey*' OR 'self-report*' OR 'dietary intake*' OR 'diet intake*' OR '24-h dietary recall*' OR '24-hour dietary recall*' OR '24-hour diet recall*' OR '24-hour recall*' OR 'food frequency questionnaire*' OR 'FFQ*' OR 'food weighing' OR 'weighed food*' OR 'feeding stud*' OR 'controlled diet*' OR 'control diet*'):ab,ti,kw |  |
| #4 | 'human'/de |  |
| #5 | [adolescent]/lim OR [adult]/lim OR [aged]/lim OR [child]/lim OR [middle aged]/lim OR [preschool]/lim OR [school]/lim OR [very elderly]/lim OR [young adult]/lim |  |
| #6 | 'article'/it OR 'article in press'/it OR 'chapter'/it OR 'conference paper'/it OR 'editorial'/it OR 'letter'/it OR 'note'/it OR 'preprint'/it OR 'review'/it OR 'short survey'/it |  |
| #7 | 1951:py OR 1952:py OR 1953:py OR 1954:py OR 1955:py OR 1956:py OR 1957:py OR 1958:py OR 1959:py OR 1960:py OR 1961:py OR 1962:py OR 1963:py OR 1964:py OR 1965:py OR 1966:py OR 1967:py OR 1968:py OR 1969:py OR 1970:py OR 1971:py OR 1972:py OR 1973:py OR 1974:py OR 1975:py OR 1976:py OR 1977:py OR 1978:py OR 1979:py OR 1980:py OR 1981:py OR 1982:py OR 1983:py OR 1984:py OR 1985:py OR 1986:py OR 1987:py OR 1988:py OR 1989:py OR 1990:py OR 1991:py OR 1992:py OR 1993:py OR 1994:py OR 1995:py OR 1996:py OR 1997:py OR 1998:py OR 1999:py OR 2000:py OR 2001:py OR 2002:py OR 2003:py OR 2004:py OR 2005:py OR 2006:py OR 2007:py OR 2008:py OR 2009:py OR 2010:py OR 2011:py OR 2012:py OR 2013:py OR 2014:py OR 2015:py OR 2016:py OR 2017:py OR 2018:py OR 2019:py OR 2020:py OR 2021:py OR 2022:py OR 2023:py |  |
| #8 | #1 AND #2 AND #3 AND #4 AND #5 AND #6 AND #7 |  |

**Web of Science via** [**https://www.webofscience.com**](https://www.webofscience.com/) (Search date: 5/12/2024; 745 results)

| #1 | "potassium" |  |
| --- | --- | --- |
| #2 | "biomarker*" OR "urine*" OR "urinary" |  |
| #3 | "diet survey*" OR "dietary survey*" OR "diet record*" OR "dietary record*" OR "nutrition survey*" OR "nutritional survey*" OR "self-report*" OR "dietary intake*" OR "diet intake*" OR "24-h dietary recall*" OR "24-hour dietary recall*" OR "24-hour diet recall*" OR "24-hour recall*" OR "food frequency questionnaire*" OR "FFQ*" OR "food weighing" OR "weighed food*" OR "feeding stud*" OR "controlled diet*" OR "control diet*" |  |
| #4 | #1 AND #2 AND #3 |  |
|  | Filter by publication years: 1950 to 2023 |  |

**Scopus via** [**https://www.scopus.com**](https://www.scopus.com) (Search date: 5/12/2024; 1113 results)

| #1 | TITLE-ABS-KEY ("potassium") |  |
| --- | --- | --- |
| #2 | TITLE-ABS-KEY ("biomarker*" OR "urine*" OR "urinary") |  |
| #3 | TITLE-ABS-KEY ("diet survey*" OR "dietary survey*" OR "diet record*" OR "dietary record*" OR "nutrition survey*" OR "nutritional survey*" OR "self-report*" OR "dietary intake*" OR "diet intake*" OR "24-h dietary recall*" OR "24-hour dietary recall*" OR "24-hour diet recall*" OR "24-hour recall*" OR "food frequency questionnaire*" OR "FFQ*" OR "food weighing" OR "weighed food*" OR "feeding stud*" OR "controlled diet*" OR "control diet*") |  |
| #4 | #1 AND #2 AND #3 |  |
|  | Filter by publication date: 1950/1/1 to 2023/12/31 |  |
|  | Filter by keyword: Human |  |

**Supplemental Table S3.**

A list of a pre-defined set of articles used to validate our search strategy.

Paradis F, Lamarche B, Robitaille J, Couillard C, Lafrenière J, Tremblay AJ, et al. Validation of an automated self-administered 24-hour dietary recall web application against urinary recovery biomarkers in a sample of French-speaking adults of the province of Québec, Canada. Appl Physiol Nutr Metab. 2022;47:173–82.

Al-Shaar L, Yuan C, Rosner B, Dean SB, Ivey KL, Clowry CM, et al. Reproducibility and Validity of a Semiquantitative Food Frequency Questionnaire in Men Assessed by Multiple Methods. Am J Epidemiol. 2021;190:1122–32.

Va P, Dodd KW, Zhao L, Thompson-Paul AM, Mercado CI, Terry AL, et al. Evaluation of measurement error in 24-hour dietary recall for assessing sodium and potassium intake among US adults - National Health and Nutrition Examination Survey (NHANES), 2014. Am J Clin Nutr. 2019;109:1672–82.

Greenwood DC, Hardie LJ, Frost GS, Alwan NA, Bradbury KE, Carter M, et al. Validation of the Oxford WebQ Online 24-Hour Dietary Questionnaire Using Biomarkers. Am J Epidemiol. 2019;188:1858–67.

Yuan C, Spiegelman D, Rimm EB, Rosner BA, Stampfer MJ, Barnett JB, et al. Relative Validity of Nutrient Intakes Assessed by Questionnaire, 24-Hour Recalls, and Diet Records as Compared With Urinary Recovery and Plasma Concentration Biomarkers: Findings for Women. Am J Epidemiol. 2018;187:1051–63.

Freedman LS, Commins JM, Moler JE, Willett W, Tinker LF, Subar AF, et al. Pooled results from 5 validation studies of dietary self-report instruments using recovery biomarkers for potassium and sodium intake. Am J Epidemiol. 2015;181:473–87.

Huang Y, Van Horn L, Tinker LF, Neuhouser ML, Carbone L, Mossavar-Rahmani Y, et al. Measurement error corrected sodium and potassium intake estimation using 24-hour urinary excretion. Hypertension. 2014;63:238–44.

Olafsdottir AS, Thorsdottir I, Gunnarsdottir I, Thorgeirsdottir H, Steingrimsdottir L. Comparison of women’s diet assessed by FFQs and 24-hour recalls with and without underreporters: associations with biomarkers. Ann Nutr Metab. 2006;50:450–60.

Charlton KE, Steyn K, Levitt NS, Zulu JV, Jonathan D, Veldman FJ, et al. Ethnic differences in intake and excretion of sodium, potassium, calcium and magnesium in South Africans. Eur J Cardiovasc Prev Rehabil. 2005;12:355–62.

Espeland MA, Kumanyika S, Wilson AC, Reboussin DM, Easter L, Self M, et al. Statistical issues in analyzing 24-hour dietary recall and 24-hour urine collection data for sodium and potassium intakes. Am J Epidemiol. 2001;153:996–1006.

Sasaki S, Yanagibori R, Amano K. Validity of a self-administered diet history questionnaire for assessment of sodium and potassium: comparison with single 24-hour urinary excretion. Jpn Circ J. 1998;62:431–5.

Bingham SA, Gill C, Welch A, Cassidy A, Runswick SA, Oakes S, et al. Validation of dietary assessment methods in the UK arm of EPIC using weighed records, and 24-hour urinary nitrogen and potassium and serum vitamin C and carotenoids as biomarkers. Int J Epidemiol. 1997;26 Suppl 1:S137–51.

**Supplemental Table S4.**

Data extraction form

| Authors | Year | Country | Age | % women | Race/ethnicity | Definition of CKD | Comorbidities | Medication use that may affect urinary potassium excretion | Number of 24-hour urine collection | Method that evaluate the completeness of 24-hour urine collection |
| --- | --- | --- | --- | --- | --- | --- | --- | --- | --- | --- |
|  |  |  |  |  |  |  |  |  |  |  |
|  |  |  |  |  |  |  |  |  |  |  |
|  |  |  |  |  |  |  |  |  |  |  |

| How potassium intake was estimated from urinary data | Dietary measure | | | | | | Primary outcome (measure of association) | Location of the results (i.e. Table 2) | Sample size |
| --- | --- | --- | --- | --- | --- | --- | --- | --- | --- |
|  | Diet/food record | 24-hour recall | Food frequency questionnaire | Diet history | Duplicate | Others |  |  |  |
|  |  |  |  |  |  |  |  |  |  |
|  |  |  |  |  |  |  |  |  |  |
|  |  |  |  |  |  |  |  |  |  |

| Outcome  (unadjusted) | Outcome (adjusted) | Confounders/covariates included in a multivariable model | Mean or median potassium intake from urinary measure | Mean or median potassium intake from dietary measure | Mean or median potassium density (potassium intake/energy intake) from dietary measure | Comments |
| --- | --- | --- | --- | --- | --- | --- |
|  |  |  |  |  |  |  |
|  |  |  |  |  |  |  |
|  |  |  |  |  |  |  |

**Supplementary Table S5.**

Joanna Briggs Institute critical appraisal checklist for analytical cross-sectional studies

|  | Yes | No | Unclear | Not applicable | Comments |
| --- | --- | --- | --- | --- | --- |
| 1. Were the criteria for inclusion in the sample clearly defined? | □ | □ | □ | □ |  |
| 1. Were the study subjects and the setting described in detail? | □ | □ | □ | □ |  |
| 1. Was the exposure measured in a valid and reliable way? | □ | □ | □ | □ |  |
| 1. Were objective, standard criteria used for measurement of the condition? | □ | □ | □ | □ |  |
| 1. Were confounding factors identified? | □ | □ | □ | □ |  |
| 1. Were strategies to deal with confounding factors stated? | □ | □ | □ | □ |  |
| 1. Were the outcomes measured in a valid and reliable way? | □ | □ | □ | □ |  |
| 1. Was appropriate statistical analysis used? | □ | □ | □ | □ |  |

Overall appraisal: Include □ Exclude □ Seek further info □
